# Frequency, patient characteristics, and clinical management for extravasation with docetaxel: a descriptive study using a large Japanese medical claims database

**DOI:** 10.1186/s40780-025-00509-3

**Published:** 2025-11-25

**Authors:** Rina Yanagisawa, Ryo Inose, Yuichi Muraki

**Affiliations:** Laboratory of Clinical Pharmacoepidemiology, Kyoto Pharmaceutical University, 5 Misasagi Nakauchi-Cho, Yamashina-Ku, Kyoto, 607-8414 Japan

**Keywords:** Extravasation, Docetaxel, Steroid, Database, Japan

## Abstract

**Background:**

Extravasation (EV) as an adverse effect of chemotherapy can induce tissue damage. Japanese guidelines on EV associated with cancer drug therapy were published in December 2022. However, few large-scale investigations on the frequency and patient clinical characteristics of EV have been conducted. In addition, although current guidelines newly recommend topical steroid use and consultation with dermatologists or plastic surgeons, it is necessary to clarify the actual status of EV management prior to the guideline revision in order to evaluate compliance with the current recommendations in the future. However, the real-world status has not been clarified to date. This study aimed to investigate the frequency of EV, the characteristics of patients who experienced EV, and the management of EV associated with docetaxel (DTX), a vesicant anticancer agent, using a large Japanese medical claims database.

**Methods:**

Patients with cancer who received DTX monotherapy between 2008 and 2020 were evaluated. The patients were identified from the hospital-based medical claims database of Medical Data Vision Co., Ltd. The baseline characteristics of the study patients, the characteristics of patients who experienced EV, steroid prescriptions, and consultations with dermatologists or plastic surgeons were descriptively investigated.

**Results:**

Among the 46,698 patients evaluated, 0.075% (35) patients developed DTX-related EV. In patients who experienced EV, 8.6% (3/35) had a history of EV. This proportion was higher than that of the overall study population. Only 34.3% (12/35) of the patients were prescribed injectable or topical steroids for EV, and 11.4% (4/35) received a consultation from the dermatology or plastic surgery department.

**Conclusions:**

DTX monotherapy–related EV occurs in 0.075% of cancer patients. The proportion of patients with a history of EV was higher among those who experienced EV than among the overall study population. Only a small proportion of patients who develop EV are prescribed topical steroids or receive consultation from the dermatology or plastic surgery department. The incidence and management of EV may change following the publication of the 2023 edition of the Japanese EV guidelines, and thus, further investigation is warranted.

**Supplementary information:**

The online version contains supplementary material available at 10.1186/s40780-025-00509-3.

## Background

Cancer is the leading cause of death in Japan, accounting for approximately 380,000 deaths annually [1, 2]. There are several treatment options for cancer, including surgical therapy, chemotherapy, and radiation therapy. Among these, chemotherapy is a primary treatment modality used across various cancer types and stages.

Chemotherapy is associated with not only systemic adverse events (e.g., nausea and vomiting, myelosuppression, and peripheral neuropathy), but also local adverse events (e.g., hair loss, constipation, and extravasation [EV]). Among these, EV is an important adverse event that occurs when anticancer agents leak out of blood vessels during administration, causing tissue damage and negatively affecting treatment outcomes and patient quality of life [3]. Anticancer agents are classified as vesicants, irritants, or non-vesicants based on their potential to cause tissue damage [4]. Among these, vesicant anticancer agents require particularly careful management, as they can cause severe tissue damage and necrosis at EV onset.

In Europe, guidelines on risk factors and management strategies for EV have been published in order to manage EV appropriately [5]. In Japan, the first guidelines of EV were published in 2009 [6], and subsequently revised [7], with the 2023 version being the latest update [4]. According to the guidelines, where 0.1–6.5% of patients treated with vesicant anticancer agents develop EV [4]. Risk factors include older age, small blood vessels, and a history of treatment with multiple anticancer agents [4, 5]. However, the documented incidence was determined based on reports from single-center studies or small case series and has not been investigated on a large scale. In addition, these results are based on multiple anticancer agents, and the occurrence of EV following anticancer monotherapy remains unclear. Furthermore, since it is a rare adverse effect, the clinical backgrounds and characteristics of patients affected by EV have not been sufficiently reported.

Both injectable and topical steroids have been traditionally used in the real world [8]. However, European guidelines do not recommend injectable steroids for EV, and there are no clear recommendations for topical steroids [5]. In Japan, the most recent revisions, published in 2023 [4], no longer recommend injectable steroids owing to insufficient evidence of their efficacy. Strong or higher topical steroids and consultation with dermatologists or plastic surgeons are recommended instead. To evaluate compliance with the current guidelines in the future, it is important to clarify the management of EV prior to the guideline revision. However, the actual injectable and topical steroid prescriptions and consultations with dermatologists or plastic surgeons in the real world before the guideline revision are unclear.

The use of real-world data (RWD) derived from clinical practice and collected from various sources has increased. Among RWD, medical claims data have been used in many studies because they include information on the diagnosis and treatment of a large number of patients at many institutions [9, 10]. Medical claims data also include prescription medications, making it possible to evaluate prescription rates and the clinical characteristics of individual patients. However, it is difficult to identify the causative agent of EV-related unwanted effects in patients who were treated with multiple anticancer agents. Furthermore, many cases are required to detect a rare adverse event such as EV.

Docetaxel (DTX), a vesicant anticancer agent, is approved for single-agent use, which allows for the clear identification of agent-specific EV events. In addition, its widespread use for multiple cancer types and various treatment lines allows for the inclusion of a large number of patients. This study, focusing on DTX monotherapy for cancer, aimed to investigate the incidence of EV, to determine the characteristics of affected patients, and to clarify steroid prescriptions and consultations with the dermatology or plastic surgery department, using data from a large Japanese medical claims database.

## Methods

### Data source

This study used a large-scale hospital-based medical claims database provided by Medical Data Vision Co., Ltd [11]. This database includes data from approximately 45% of the 1,746 Diagnosis Procedure Combination (DPC) hospitals in Japan, covering a cumulative total of approximately 34.51 million patients. A key feature of this database is its extensive coverage of older adults, with 48% of registered patients being 65 years of age or older. Information such as the diagnosis, medications, and procedures for each patient can be obtained from the database.

### Patients

Patients who received DTX between April 1, 2008, and December 31, 2020 were included in this study. To investigate the background of the study participants, patients without data for the 6 months prior to their first DTX administration were excluded. To evaluate the EV related to DTX monotherapy, patients who were administered other anticancer agents during the DTX treatment period were excluded. Furthermore, patients with no cancer diagnosis prior to the first administration of DTX and those who experienced EV within 1 month before the first administration of DTX were excluded.

The DTX treatment period was defined as the interval between the first and last administration of DTX. If there was an interval of more than 30 days between administrations, it was not considered a consecutive treatment period, and the interval data were excluded.

### Variable definitions

EV was identified using International Classification of Diseases 10th Revision (ICD-10) codes T451 (anticancer agent leakage skin disorder) and T509 (agent leakage skin disorder). Only the first EV was included for patients with multiple EVs. Cancer type was determined using ICD-10 codes (Supplementary Table 1). Meanwhile, the anticancer agents used were identified using the Anatomical Therapeutic Chemical (ATC) classification of World Health Organization (Supplementary Table 2) and classified according to their ability to cause tissue damage upon EV, based on Japanese guidelines [4]. Diabetes mellitus was defined by a combination of claims codes (Supplemental Table 3) and therapeutic agents based on the ATC classification (Supplemental Table 2), in accordance with a previously published validation study [12]. The use of a central venous access route was identified using claims codes (Supplemental Table 3). Cancer type was defined as a definitive diagnosis of cancer immediately before the first administration of DTX, and the history of anticancer agent administration, diabetes mellitus, and EV were investigated retrospectively for 6 months prior to the first administration of DTX.

The present study focused on immediate-onset EV. The management of EV was defined as any treatment administered on the day of the event’s onset. EV diagnosis is recorded only on a monthly basis, making it impossible to pinpoint the exact onset date. Therefore, the DTX administration date within the same month was substituted as the index date for this study, assuming EV onset occurred on that day. Prescriptions for injectable or topical steroids and consultations with dermatologists or plastic surgeons were defined as having been conducted on the index date. Steroid was defined based on the ATC classification of World Health Organization (Supplementary Table 2). Dexamethasone and triamcinolone injections were excluded from the study because they were likely to be used for purposes other than EV treatment. Consultation with the dermatology or plastic surgery department was identified as department codes 130 for plastic surgery and 300 for dermatology) in the medical records.

### Statistical analysis

The baseline characteristics of the study cohort, the clinical characteristics of patients who experienced EV, the details of steroid prescriptions, and the consultations with the dermatology or plastic surgery department for EV were descriptively analyzed. Stata MP 17 (Stata Corp., College Station, TX, USA) was used.

## Results

### Baseline characteristics of the study population

**Fig. 1:**
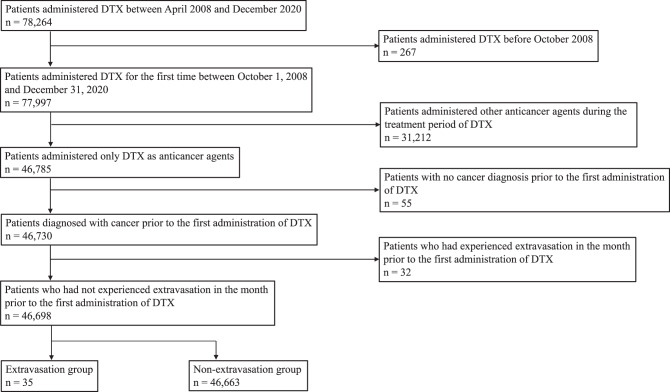
Patient selection flowchart. DTX: docetaxel

A total of 46,698 patients with cancer who received DTX monotherapy during the study period were evaluated. The patient selection flowchart is shown in Fig. 1. EV occurred in 35 patients, yielding an incidence rate of 0.075%. The characteristics for the 46,698 patients who received DTX monotherapy between October 2008 and December 2020 are shown in Table 1. The median age was 66 years, and 22,868 patients (49.0%) were male. A history of EV was present in 21 patients (0.04%), and a central venous access was present in 1,010 patients (2.2%). A history of diabetes mellitus was documented in 4,717 patients (10.1%), and 26,441 patients (56.6%) had a history of anticancer agent administration.

**Table 1:** Characteristics of patients who received DTX monotherapy between October 2008 and December 2020

	Target patients (n = 46,698)	
Age (years)^1^	66	[57–73]
Sex^2^		
Male	22,868	(49.0)
Cancer base hospital^2^	40,037	(85.7)
Number of hospital beds^2^		
≤199	1,025	(2.2)
200–499	22,767	(48.8)
≥500	22,906	(49.1)
CCI^1^	5	[2–9]
History of EV^2^	21	(0.04)
Central venous access^2^	1,010	(2.2)
History of diabetes mellitus^2^	4,717	(10.1)
History of anticancer agent administration^2^	26,441	(56.6)
Cancer types targeted by DTX administration^2^		
Breast	15,914	(34.1)
Lung	14,327	(30.7)
Prostate	5,209	(11.2)
Esophageal	4,067	(8.7)
Stomach	3,190	(6.8)
Head and neck	2,276	(4.9)
Ovarian	1,444	(3.1)
Uterine corpus	1,251	(2.7)
Others	1,284	(2.7)

^1^Data are presented as median [interquartile range]

^2^Data are presented as n (%)

CCI: Charlson Comorbidity Index, DTX: docetaxel, EV: extravasation

## Characteristics of patients with EV

The characteristics of the 35 patients who developed EV during DTX monotherapy are presented in Table 2. A history of EV was reported in 8.6% (3/35), and 2.9% (1/35) had central venous access. Furthermore, 5.7% (2/35) of the patients had a history of diabetes mellitus, and a history of anticancer agent administration, many of which were vesicant agents, was documented in 62.9% (22/35).

**Table 2:** Baseline characteristics of the 35 patients who developed EV

Sex (M/F)	Age (years)	Cancer base hospital	Number of hospital beds	CCI	History of EV	Central venous access	History of diabetes mellitus	History of anticancer agent administration	Previously administered anticancer agents	Classification of anticancer agents according to their ability to cause local damage after EV	Cancer types targeted by DTX administration	Patient no.
F	56	Yes	≥500	9	Yes	No	Yes	Yes	Epirubicin Cyclophosphamide	Vesicant Irritant	Breast	1
F	65	Yes	200–499	2	Yes	No	No	Yes	Doxorubicin Cisplatin	Vesicant No report	Uterine corpus	2
F	56	Yes	≥500	2	Yes	No	No	Yes	Paclitaxel Carboplatin	Vesicant Irritant	Uterine corpus	3
F	69	Yes	≥500	3	No	Yes	No	No	–	–	Esophageal	4
F	71	Yes	≥500	8	No	No	No	Yes	Doxorubicin Cyclophosphamide	Vesicant Irritant	Breast	5
M	63	Yes	200–499	2	No	No	No	No	–	–	Stomach	6
F	38	Yes	≥500	2	No	No	No	Yes	Epirubicin Cyclophosphamide	Vesicant Irritant	Breast	7
F	35	Yes	≥500	2	No	No	No	Yes	Epirubicin Cyclophosphamide	Vesicant Irritant	Breast	8
F	80	Yes	200–499	9	No	No	No	No	–	–	Breast	9
M	70	Yes	200–499	3	No	No	No	No	–	–	Prostate	10
F	61	Yes	≥500	8	No	No	No	No	–	–	Breast	11
F	56	Yes	200–499	2	No	No	No	Yes	Doxorubicin Cyclophosphamide	Vesicant Irritant	Breast	12
M	66	Yes	≥500	8	No	No	No	No	–	–	Prostate	13
M	73	Yes	≥500	9	No	No	No	No	–	–	Prostate	14
F	46	Yes	200–499	2	No	No	No	No	–	–	Breast	15
F	52	Yes	≥500	2	No	No	No	Yes	Epirubicin Cyclophosphamide	Vesicant Irritant	Breast	16
M	59	Yes	≥500	11	No	No	No	Yes	Amrubicin	Vesicant	Other	17
F	76	Yes	≥500	10	No	No	No	Yes	Paclitaxel Bevacizumab	Vesicant Nonvesicant	Breast	18
F	62	Yes	≥500	2	No	No	No	Yes	Paclitaxel Carboplatin	Vesicant Irritant	Uterine corpus	19
F	69	Yes	≥500	3	No	No	Yes	No	–	–	Uterine corpus	20
F	66	Yes	≥500	8	No	No	No	Yes	Epirubicin Cyclophosphamide	Vesicant Irritant	Breast	21
F	25	Yes	200–499	2	No	No	No	Yes	Epirubicin Cyclophosphamide	Vesicant Irritant	Breast	22
M	47	Yes	200–499	2	No	No	No	Yes	Fluorouracil Cisplatin	Irritant No report	Esophageal	23
F	44	Yes	≥500	2	No	No	No	Yes	Epirubicin Fluorouracil Cyclophosphamide	Vesicant Irritant Irritant	Breast	24
M	75	Yes	≥500	9	No	No	No	No	–	–	Esophageal	25
F	87	Yes	≥500	2	No	No	No	No	–	–	Esophageal	26
M	74	Yes	≥500	3	No	No	No	No	–	–	Esophageal	27
F	71	Yes	≥500	10	No	No	No	Yes	Pemetrexed	Nonvesicant	Lung	28
F	80	Yes	≥500	10	No	No	No	Yes	Paclitaxel Carboplatin Bevacizumab	Vesicant Irritant Nonvesicant	Ovarian	29
F	41	Yes	200–499	2	No	No	No	Yes	Paclitaxel Carboplatin	Vesicant Irritant	Uterine corpus	30
M	79	Yes	≥500	9	No	No	No	No	–	–	Lung	31
M	61	Yes	≥500	8	No	No	No	Yes	Pemetrexed Cisplatin	Nonvesicant No report	Lung	32
M	47	Yes	≥500	8	No	No	No	Yes	Carboplatin Pembrolizumab Pemetrexed	Irritant Nonvesicant Nonvesicant	Lung	33
F	40	Yes	200–499	2	No	No	No	Yes	Epirubicin Cyclophosphamide	Vesicant Irritant	Breast	34
M	64	Yes	≥500	9	No	No	No	Yes	Paclitaxel Carboplatin Atezolizumab	Vesicant Irritant Nonvesicant	Lung	35

CCI:Charlson Comorbidity Index, DTX: docetaxel, EV: extravasation, M/F: male/female

## Clinical characteristics of EV events

The clinical characteristics of the 35 patients who developed EV are shown in Table 3. Regarding EV events, 51.4% (18/35) occurred during the first DTX administration. Following the onset of EV, DTX was suspended or discontinued in 60.0% (21/35) of the patients. A consultation with the dermatology or plastic surgery department on the index date was provided to 11.4% (4/35) of these patients.

**Table 3:** Clinical characteristics of the 35 EV events

Consultation with dermatology or plastic surgery department	Prescription for injectable or topical steroids	Number of DTX administrations until EV occured	Interruption of DTX administration after EV	Patient no.
Yes	–	2	No	18
Yes	–	2	Yes	29
Yes	Hydrocortisone sodium succinate Clobetasol propionate	2	Yes	35
Yes	–	4	No	9
No	Hydrocortisone sodium succinate Betamethasone valerate	1	No	5
No	Hydrocortisone sodium succinate Diflorasone diacetate	1	Yes	8
No	Hydrocortisone sodium succinate Clobetasol propionate	1	No	10
No	–	1	No	11
No	–	1	No	13
No	–	1	Yes	4
No	–	1	No	16
No	–	1	No	19
No	–	1	Yes	20
No	–	1	Yes	1
No	Hydrocortisone sodium succinate Betamethasone butyrate propionate	1	Yes	22
No	–	1	No	2
No	–	1	No	24
No	–	1	Yes	25
No	–	1	Yes	26
No	–	1	Yes	27
No	Hydrocortisone sodium succinate Clobetasol propionate	1	Yes	30
No	–	1	Yes	3
No	Betamethasone butyrate propionate	2	Yes	6
No	–	2	No	7
No	–	2	No	14
No	Hydrocortisone sodium succinate Clobetasol propionate	2	Yes	32
No	–	3	No	12
No	–	3	Yes	23
No	Clobetasol propionate	3	Yes	31
No	–	3	No	33
No	Hydrocortisone sodium phosphate	4	Yes	15
No	–	4	Yes	21
No	Betamethasone sodium phosphate Clobetasol propionate	4	Yes	34
No	Clobetasol propionate	8	Yes	17
No	–	10	Yes	28

DTX: docetaxel, EV: extravasation

## Prescription of injectable and topical steroids for EV

The prescriptions for injectable and topical steroids on the index date are shown in Table 4. In total, 34.3% (12/35) in the EV group were prescribed topical or injectable steroids. Among them, 8.3% (1/12) patients were prescribed injectable steroids only, while 25.0% (3/12) patients were prescribed topical steroids only. The remaining 66.7% (8/12) patients were prescribed both injectable and topical steroids. All prescribed topical steroids were strong or higher.

**Table 4:** Prescriptions for injectable and topical steroids

Prescription details		Number of patients
Injectable only		**1**
	Hydrocortisone sodium phosphate	1
Topical only		**3**
	Clobetasol propionate	2
	Betamethasone butyrate propionate	1
Injectable + topical		**8**
	Hydrocortisone sodium succinate	
	+ Clobetasol propionate	4
	+ Betamethasone valerate	1
	+ Diflorasone diacetate	1
	+ Betamethasone butyrate propionate	1
	Betamethasone sodium phosphate	
	+ Clobetasol propionate	1
Total		**12**

## Discussion

EV during chemotherapy negatively affects the treatment outcomes and patient quality of life [3]. However, the frequency, patient characteristics, and treatments have not been elucidated. In the current study, 0.075% of cancer patients treated with DTX monotherapy developed EV. Compared with the entire target population, patients who experienced EV more often had a history of EV, and less than half of the patients who developed EV were prescribed injectable or topical steroids.

In this study, the frequency of EV in patients administered with DTX monotherapy was 0.075%. Previously, the frequency of EV caused by vesicant anticancer agents has been reported to be 0.1–6.5% [4]. Although the patient characteristics, target anticancer agents, and research methods in this study differed from those in previous reports, the results were comparable to those in previous reports. We have determined for the first time the frequency of EV in a large population of patients treated with DTX monotherapy. These results can be used to compare the frequency of EV in different centers and may be useful in the evaluation of EV countermeasures in each institution. On the other hand, drug adverse events tend to be coded using ICD codes only when they become the chief complaint or a serious clinical finding, and it has been pointed out that clinical symptoms are underestimated [13]. Therefore, mild EV that do not require prescriptions or tests are less likely to be registered as diagnostic records, and the frequency of EV in this study may be underestimated.

In this study, subsequent DTX administration was suspended or discontinued in 21 of the 35 patients who experienced EV. These treatment interruptions may have been influenced by the EV events, suggesting that EV represents a considerable challenge to therapy continuation. Furthermore, a history of EV was reported in 8.6% (3/35) of patients. This highlights that healthcare providers should confirm a patient’s history of EV before starting DTX monotherapy and administer the treatment with caution. However, these results should be interpreted with caution. Patients with a history of EV might have been monitored more closely, which may have led to a higher likelihood of a subsequent EV being diagnosed and reported.

This study has several important implications. EV occurred even in patients with central venous access. Previous reports indicate that the incidence of EV from central venous devices ranges from 0.4–4.7% [4]. The present study showed a similar trend, suggesting the need for caution regarding EV, irrespective of the administration route. Furthermore, more than half of the patients developed EV during their initial DTX administration. These findings underscore the importance of vigilant monitoring during agent administration, starting with the very first infusion. Among patients with a history of anticancer agent administration, the majority had been treated with vesicant agents. Basic research suggests that the mechanisms of endothelial damage differ between classes of vesicant agents, such as anthracyclines and taxanes [14, 15]. However, whether these agent effects translate to long-term vascular fragility in clinical real-world settings has not been sufficiently verified, and studies directly comparing EV risks based on the type of previously administered anticancer agent are scarce. This area warrants further investigation.

Previous reports have identified old age, history of diabetes mellitus, and history of anticancer agent administration as risk factors for EV [4, 5]. However, the proportion of patients with a history of diabetes mellitus was lower in the EV group than that of the overall study population (5.7% vs 10.1%). Furthermore, age and history of anticancer agent administration were equivalent in the EV group and the overall study population. The degree of vascular damage varies in diabetes mellitus [16]. The possibility that differences in the severity of diabetes mellitus influenced the results cannot be excluded; however, this could not be evaluated in this study. Although age and history of anticancer agent administration are listed as risk factors in current guidelines [4, 5], the underlying patient characteristics are not specified. This lack of details precluded a direct comparison of our findings to investigate these discrepancies.

A total of 34.3% (12/35) of the patients with EV in the current study were prescribed injectable or topical steroids for EV. In addition, 11.4% (4/35) of the patients in the EV group received dermatology or plastic surgery consultations. During the study period, these treatments were not explicitly recommended in Japanese guidelines [6, 7]. Therefore, these results do not indicate non-compliance with the latest guidelines [4] but rather reflect clinical practice at the time. Guidelines in other countries recommend urgent referral to plastic surgeons for severe EV [5, 17, 18], and the new Japanese guidelines [4] conform to this international standard. However, there are no clear recommendations for the use of topical steroids in other international guidelines [5, 17, 18], whereas Japan’s new guidelines recommend strong or higher topical steroids, demonstrating a high degree of individuality [4]. The use of topical steroids is expected to increase in Japan based on the new recommendation; therefore, attention should be paid to the trends in their use. Additionally, healthcare providers should ensure that patients receive consultation from dermatologists or plastic surgeons and that the prescription, efficacy, and safety of topical steroids are continuously evaluated.

This study has limitations. First, the definition and identification of EV present several challenges. EV was identified using ICD-10 codes, but these codes have not been validated for accurately identifying true EV events. EV is a rare adverse event, making it difficult to accumulate a sufficient number of cases for a formal validation study, even in a multicenter study. Due to this constraint, our study relied solely on ICD-10 codes to identify EV cases. Furthermore, data on less severe EV may not have been registered in the DPC database, possibly underestimating the frequency of EV. This predicament underscores the need for better alignment between clinical diagnoses and billing codes. Second, because this study utilized a hospital-based claims database, it was not possible to track medical information from different institutions for the same patient. This may have affected the patients’ medical history. Furthermore, since the exact date of EV onset could not be identified, the DTX administration date within the same month as EV onset was used as index date. Therefore, this index date may differ from the actual date of EV onset. Additionally, it was not possible to obtain information on the severity of the disease. The consultations with the dermatology or plastic surgery department shown in these results may be affected by multiple factors beyond EV severity, including the availability of access mechanisms and triage systems at each center. Therefore, these results do not directly indicate EV severity. Finally, the influence of unknown confounding factors cannot be ruled out. Despite these limitations, to the best of our knowledge, this is the first study to evaluate EV using a large medical claims database, providing useful information for administering DTX for cancer. The use of the large medical claims database provided a method for evaluating a rare chemotherapy-related adverse event such as EV.

## Conclusions

In total, 0.075% of cancer patients develop EV during DTX monotherapy. Patients who experienced EV were more likely to have a history of EV than the overall study population. Topical steroids are prescribed only to a small proportion of patients, and only few patients receive dermatology or plastic surgery consultations. The incidence and management of EV may change with the publication of the 2023 guidelines, and thus, further investigation is warranted.

## Electronic supplementary material

Below is the link to the electronic supplementary material.

Supplementary Material 1

Supplementary Material 2

Supplementary Material 3

## Data Availability

The data that support the findings of this study are available from MDV but restrictions apply to the availability of these data, which were used under license for the current study, and so are not publicly available.

## Abbreviations

ATC: Anatomical Therapeutic Chemical
DPC: Diagnosis Procedure Combination
DTX: docetaxel
EV: extravasation
ICD-10: International Classification of Diseases, 10th Revision
RWD: real-world data

## References

[1] Ministry of Health. Labour and welfare. Overview of Vital Statistics in 2021. 2022. Accessed 5 May 2025. https://www.mhlw.go.jp/toukei/saikin/hw/jinkou/kakutei21/dl/10_h6.pdf

[2] National Cancer Center, Japan. Latest Cancer Statistics. 2024. Accessed 5 May 2025. https://ganjoho.jp/reg_stat/statistics/stat/summary.html

[3] Guo Y, Huo J. Salvage treatment after extravasation of chemotherapy agents: A rare case report. Asian J Surg. 2023;46:2750–51. 36658002 10.1016/j.asjsur.2023.01.022

[4] Matsumoto K, Ryushima Y, Sato J, Aizawa Y, Aoyama T, Akaishi Y, et al. Extravasation associated with cancer drug therapy: multidisciplinary guideline of the Japanese Society of Cancer Nursing. ESMO Open. 2024;9:103932. 39389005 10.1016/j.esmoop.2024.103932 PMC11490930

[5] Pérez Fidalgo JA, García Fabregat L, Cervantes A, Margulies A, Vidall C, Roila F. On behalf of the ESMO Guidelines Working Group. Management of chemotherapy extravasation: ESMO-EONS Clinical Practice Guidelines. Ann Oncol. 2012;23:167–73. 10.1093/annonc/mds294 22997449

[6] Cancer Chemotherapy Nursing Working Group at St. Luke’s College of Nursing. 2009 Guideline for Chemotherapy Nursing of Cancer Outpatients: Prevention and early detection of extravasation of carcinostatic agent and measures, 1st version. Tokyo: KANEHARA & CO., LTD; 2009.

[7] Japanese Society of Cancer Nursing. 2014 Guideline for Chemotherapy Nursing of Cancer Outpatients: Prevention and early detection of extravasation of carcinostatic agent and device-related complication and measures, 2nd version. Tokyo: KANEHARA & CO., LTD; 2014.

[8] Mitsuma A, Sawaki M, Shibata T, Morita S, Inada M, Shimokata T, et al. Extravasation of pegylated-liposomal doxorubicin: favorable outcome after immediate subcutaneous administration of corticosteroids. Nagoya J Med Sci. 2012;74:189–92. 22515126 PMC4831265

[9] Ito S, Goto R, Inose R, Kusama Y, Ono A, Koizumi R, et al. A study of trends and factors associated with therapeutic drug monitoring (TDM) implementation for arbekacin treatment using a large Japanese medical claims database. Infect Chemother. 2022;28:1266–72. 10.1016/j.jiac.2022.05.007 35606308

[10] Hashimoto H, Imai S, Yamashita R, Kiyomi A, Sugiura M. Association of antipsychotic drugs with the risk of recurrent venous thromboembolism: a retrospective study of data from a Japanese inpatient database. Drugs Real World Outcomes. 2023;11:109–16. 38015358 10.1007/s40801-023-00401-2 PMC10928045

[11] Medical Data Vision Co., Ltd. 2025. Accessed 5 May 2025. https://www.mdv.co.jp/

[12] Nishioka Y, Takeshita S, Kubo S, Myojin T, Noda T, Okada S, et al. Appropriate definition of diabetes using an administrative database: A cross-sectional cohort validation study. J Diabetes Investig. 2022;13:249–55. 34327864 10.1111/jdi.13641 PMC8847127

[13] Nadkarni PM. Drug safety surveillance using de-identified EMR and claims data: issues and challenges. J Am Med Inf Assoc. 2010;17:671–74. 10.1136/jamia.2010.008607 PMC3000764 20962129

[14] Nebigil CG, Désaubry L. Updates in anthracycline-mediated cardiotoxicity. Front Pharmacol. 2018;9:1262. 30483123 10.3389/fphar.2018.01262 PMC6240592

[15] Ahire C, Nyul-Toth A, DelFavero J, Gulej R, Faakye JA, Tarantini S, et al. Accelerated cerebromicrovascular senescence contributes to cognitive decline in a mouse model of paclitaxel (Taxol)-induced chemobrain. Aging Cell. 2023;22:e13832. 37243381 10.1111/acel.13832 PMC10352561

[16] Schram MT, Henry RM, van Dijk RA, Kostense PJ, Dekker JM, Nijpels G, et al. Increased central artery stiffness in impaired glucose metabolism and type 2 diabetes. Hypertension. 2003;43:176–81. 14698999 10.1161/01.HYP.0000111829.46090.92

[17] MD Anderson Cancer Center. Extravasation management (vesicant and contrast agents). 2021. Accessed 19 June 2025. https://www.mdanderson.org/content/dam/mdanderson/documents/for-physicians/algorithms/clinical-management/clin-management-extravasation-web-algorithm.pdf

[18] NHS England. Guidelines for the management of a extravasation of a systemic anti-cancer therapy including cytotoxic agents. 2017. Accessed 19 June 2025. https://www.england.nhs.uk/midlands/wp-content/uploads/sites/46/2019/05/management-extravasation-of-a-systemic-anti-cancer-therapy-including-cytotoxic-agents.pdf

