# Supplementary material for: Frequency, patient characteristics, and clinical management for extravasation with docetaxel: a descriptive study using a large Japanese medical claims database
Source: J Pharm Health Care Sci. 2025 Nov 25;11:105. doi: 10.1186/s40780-025-00509-3 (PMC12649079; doi:10.1186/s40780-025-00509-3)
Supplement: Supplementary file 1 — Supplementary Material 1 [file 40780_2025_509_MOESM1_ESM.docx]

**Supplemental Table 1.** List of ICD-10 codes

| **ICD-10 code** | **Disease** |
| --- | --- |
| C00–14, C30–32 | Head and neck cancer |
| C15 | Esophageal cancer |
| C16 | Stomach cancer |
| C33–34 | Lung cancer |
| C50 | Breast cancer |
| C54 | Uterine corpus cancer |
| C56 | Ovarian cancer |
| C61 | Prostate cancer |
| C17–29, C35–49, C51–53, C55, C57–60, C62–97 | Other cancers |

ICD-10: International Classification of Diseases, 10th Revision
