# Supplementary material for: Frequency, patient characteristics, and clinical management for extravasation with docetaxel: a descriptive study using a large Japanese medical claims database
Source: J Pharm Health Care Sci. 2025 Nov 25;11:105. doi: 10.1186/s40780-025-00509-3 (PMC12649079; doi:10.1186/s40780-025-00509-3)
Supplement: Supplementary file 2 — Supplementary Material 2 [file 40780_2025_509_MOESM2_ESM.docx]

**Supplemental Table 2.** List of ATC codes

| **ATC code** | **Classified name** |
| --- | --- |
| L01CD02 | Docetaxel |
| H02 (excluding H02AB02 and H02AB08) | Injectable steroids |
| D07 | Topical steroids |
| L01 (excluding L01CD02) | Anticancer agent |
| A10 | Drugs used in diabetes |

ATC: Anatomical Therapeutic Chemical Classification System
