# Supplementary material for: Frequency, patient characteristics, and clinical management for extravasation with docetaxel: a descriptive study using a large Japanese medical claims database
Source: J Pharm Health Care Sci. 2025 Nov 25;11:105. doi: 10.1186/s40780-025-00509-3 (PMC12649079; doi:10.1186/s40780-025-00509-3)
Supplement: Supplementary file 3 — Supplementary Material 3 [file 40780_2025_509_MOESM3_ESM.docx]

**Supplemental Table 3.** List of receipt codes

|  | **Receipt code** | | | | |
| --- | --- | --- | --- | --- | --- |
| Central venous access | 130004410 | 130004670 | 130008510 | 130009070 | 130009170 |
|  | 130009570 | 130009870 | 130010670 | 130011170 | 130011570 |
|  | 130011610 |  |  |  |  |
| Diabetes mellitus | 2500001 | 2500013 | 2500014 | 2500015 | 2500024 |
|  | 2500027 | 2500031 | 2500037 | 2500041 | 2501002 |
|  | 2501003 | 2501005 | 2502004 | 2502006 | 2503005 |
|  | 2503007 | 2504004 | 2504005 | 2504006 | 2504010 |
|  | 2504012 | 2504013 | 2505011 | 2505018 | 2505021 |
|  | 2506006 | 2506011 | 2507025 | 2507028 | 2507029 |
|  | 2509003 | 2509004 | 2510003 | 2713009 | 2714002 |
|  | 6489003 | 7751001 | 7751002 | 7915002 | 7915003 |
|  | 8830028 | 8830029 | 8830030 | 8830031 | 8830032 |
|  | 8830033 | 8830039 | 8830040 | 8830041 | 8830042 |
|  | 8830043 | 8830044 | 8830045 | 8830405 | 8830756 |
|  | 8831132 | 8831401 | 8832747 | 8833419 | 8833420 |
|  | 8834843 | 8835244 | 8835464 | 8835685 | 8835871 |
|  | 8835941 | 8836104 | 8836563 | 8838062 | 8838063 |
|  | 8838064 | 8838065 | 8838066 | 8838067 | 8838068 |
|  | 8838069 | 8838070 | 8838071 | 8838072 | 8838073 |
|  | 8838074 | 8838075 | 8838076 | 8838077 | 8838078 |
|  | 8838079 | 8838080 | 8838081 | 8838619 | 8838621 |
|  | 8838633 | 8839324 | 8840104 | 8840710 | 8841021 |
|  | 8841679 | 8841680 | 8841681 | 8841682 | 8841683 |
|  | 8841684 | 8841685 | 8841686 | 8841687 | 8841688 |
|  | 8841689 | 8841690 | 8841691 | 8841692 | 8841693 |
|  | 8841694 | 8841695 | 8841696 | 8841697 | 8841698 |
|  | 8843105 | 8843106 | 8843120 | 8843121 | 8843122 |
|  | 8843123 | 8843124 | 8843125 | 8843126 | 8843127 |
|  | 8843128 | 8843375 | 8843376 | 8843377 | 8843378 |
|  | 8843379 | 8843380 | 8843381 | 8843382 | 8843383 |
|  | 8843388 | 8843389 | 8843390 | 8843391 | 8843392 |
|  | 8843393 | 8843394 | 8843395 | 8843396 | 8843439 |
|  | 8843448 | 8843449 | 8843450 | 8843451 | 8843452 |
|  | 8843453 | 8843454 | 8843455 | 8843456 | 8843619 |
|  | 8843620 | 8843621 | 8843622 | 8843623 | 8843624 |
|  | 8843625 | 8843626 | 8843627 | 8843982 | 8843983 |
|  | 8843984 | 8843985 | 8843986 | 8843987 | 8843988 |
|  | 8843989 | 8843990 | 8843991 | 8843992 | 8843993 |
|  | 8843994 | 8843995 | 8843996 | 8843997 | 8844022 |
|  | 8844023 | 8844024 | 8844025 | 8844026 | 8844027 |
|  | 8844028 | 8844029 | 8844030 | 8844031 | 8844045 |
|  | 8844089 | 8844233 | 8844346 | 8844347 | 8844536 |
|  | 8844537 | 8844626 | 8844627 | 8844628 | 8844629 |
|  | 8844652 | 8844653 | 8845043 | 8845044 | 8845045 |
|  | 8845046 | 8845047 | 8845048 | 8845049 | 8845050 |
|  | 8845051 | 8845052 | 8845053 | 8845054 | 8845055 |
|  | 8845056 | 8845057 | 8845058 | 8845059 | 8845060 |
|  | 8845061 | 8845062 | 8845063 | 8845064 | 8845065 |
|  | 8845066 | 8845067 | 8845068 | 8845069 | 8845070 |
|  | 8845071 | 8845072 | 8845073 | 8845074 | 8845075 |
|  | 8845076 | 8845077 | 8845078 | 8845079 | 8845080 |
|  | 8845081 | 8845082 | 8845083 | 8845084 | 8845085 |
|  | 8845086 | 8845087 | 8845088 | 8845089 | 8845090 |
|  | 8845091 | 8845092 | 8845093 | 8845094 | 8845095 |
|  | 8845096 | 8845097 | 8845098 | 8845099 | 8845100 |
|  | 8845128 | 8845198 | 8845842 | 8848108 | 8848583 |
|  | 8848632 | 8848633 | 8848634 | 8848768 | 8849056 |
|  | 8849058 | 8849181 | 8849469 | 8849470 | 8849471 |
|  | 8849472 | 8849473 | 8849474 | 8849475 | 8849476 |
|  | 8849477 | 8849478 | 8849557 | 8849558 | 8849585 |
|  | 8849586 | 8849587 | 8849588 | 8849589 | 8849590 |
|  | 8849591 | 8849592 | 8849593 | 8849594 | 8849874 |
|  | 8849976 | 8850065 |  |  |  |
